# Fatal plant poisoning cases in Tunisia: a retrospective series

**DOI:** 10.11604/pamj.2026.54.31.53132

**Published:** 2026-06-05

**Authors:** Amira Horchani, Rim Hamdi, Latifa Thabti, Jihen Ladib, Wahiba Douki

**Affiliations:** 1 Laboratory of Pharmacognosy, Faculty of Pharmacy, University of Monastir, *Rue Avicenne*, 5000 Monastir, Tunisia,; 2 Department of Pharmacy, Fattouma Bourguiba University Hospital of Monastir, *Rue du 1er Juin 1995*, 5000 Monastir, Tunisia,; 3 Laboratory of Toxicology, Fattouma Bourguiba University Hospital of Monastir, *Rue du 1er Juin 1995*, 5000 Monastir, Tunisia

**Keywords:** Poisoning, medicinal plants, toxic plants, alkaloids, mortality

## Abstract

Medicinal plants are widely used and commonly considered safe. However, certain species contain toxic compounds that may cause severe or fatal poisoning. This study aimed to describe fatal cases of plant poisoning reported to the toxicology laboratory of a Tunisian university hospital over a ten-year period. This retrospective study was conducted at the Toxicology Laboratory of Fattouma Bourguiba University Hospital in Monastir, Tunisia, over the period from 2014 to 2023. Data were collected from toxicological analysis reports and laboratory records. Only cases of suspected plant poisoning referred for medico-legal toxicological analysis and resulting in death were included. Data collected related to patient characteristics, plant species involved, circumstances of exposure, and clinical signs observed before death. Five fatal cases of plant poisoning were recorded. Most cases occurred in the context of medicinal use. In one case, the circumstances of exposure were uncertain but were reported to have followed advice from a spiritual healer. Three plant species were implicated: Peganum harmalaL., Ephedra alata Decne. subsp. alenda (Stapf) Trab and Ecballium elaterium (L.) A. Rich. Clinical manifestations ranged from gastrointestinal and neurological symptoms progressing to respiratory arrest, to cardiovascular complications and multiorgan failure, depending on the plant species. Fatal plant poisonings, although rare, highlight the potential toxicity of medicinal plants commonly perceived as safe, mainly due to the presence of toxic bioactive compounds. Increasing awareness among healthcare professionals and the general population is essential to prevent such outcomes.

## To the editors of the Pan african Medical Journal

Medicinal plants and herbal remedies have been widely used in the prevention and treatment of diseases due to their high content of bioactive compounds with therapeutic properties [1]. However, some plants contain toxic components that may cause adverse effects, ranging from mild skin irritation to severe illness or death [2]. In Tunisia, information regarding fatal plant poisoning incidents remains scarce [3]. This study aimed to describe fatal cases of plant poisoning reported to the toxicology laboratory of a Tunisian university hospital over ten years (2014-2023).

This retrospective study was conducted at the Toxicology Laboratory of Fattouma Bourguiba University Hospital in Monastir, Tunisia, over nine years from 2014 to 2023. Data were collected from toxicological analysis reports and laboratory records. Only cases of suspected plant poisoning referred for medico-legal toxicological analysis and resulting in death were included. Non-fatal plant poisoning cases were managed in clinical departments, and follow-up information was not available. Collected data included patient demographics, suspected plant species, circumstances of exposure (including mode of preparation, route of administration, and context of poisoning), and clinical signs observed before death.

During the study period, five deaths due to plant poisoning were recorded. All intoxications occurred in the context of medicinal use, except one case in which the circumstances were uncertain but were reported to have followed advice from a spiritual healer. Three plant species were implicated. Table 1 summarizes the details of each fatal intoxication, including plant species, year, patient characteristics, plant part used, intended use, and clinical outcomes. Depending on the plant species, poisoning caused either neurological and gastrointestinal symptoms or multiorgan failure.

**Table 1:** fatal cases of medicinal plant poisoning

Plant (family)	Year	Age/sex	Part used	Intended use	Clinical effects/cause of death
*Peganum harmala* L. (Nitrariaceae)	2014	42, Male	Not reported	Not reported	Gastrointestinal and neurological symptoms, progressing to respiratory arrest.
	2019	22, Female	Powdered seed	Weight loss	
	2019	37, Male	Seeds mixed with honey	Infertility	
*Ephedra alata* Decne. subsp. *alenda* (Stapf) Trab. (Ephedraceae)	2018	42, Male	Aerial parts, infusion	Rhinitis	Balance disorders. Autopsy revealed pulmonary and cerebral congestion, myocardial fatty infiltration, and purulent bronchial secretions.
*Ecballium elaterium* (L.) A. Rich. (Cucurbitaceae)	2023	27, Male	Fruit juice	Hepatitis	Multiorgan failure (liver and kidney).

The plant species implicated in the fatal poisonings reported in this study are part of the Tunisian flora and are traditionally used for their therapeutic properties. However, traditional medicinal use does not guarantee safety, as many of these plants contain complex bioactive compounds, some of which may be toxic, particularly alkaloids [4]. The seeds of *Peganum harmala* L. contain β-carboline alkaloids such as harmalol, harmaline, and harmine. These compounds have been associated with toxic effects on the neurological, gastrointestinal, and cardiovascular systems, whether following therapeutic use or overdose [5,6]. Similarly, *Ephedra* species contain sympathomimetic alkaloids, principally ephedrine and pseudoephedrine, which are linked to adverse events such as hypertension, arrhythmias, seizures, and even fatal outcomes when used improperly. These risks have led to regulatory restrictions on *Ephedra*-containing products in several countries [7,8]. The juice of *Ecballium elaterium* L. contains cucurbitacins, which are potent, highly oxygenated tetracyclic triterpenoids. These compounds are highly toxic and may cause mucosal irritation, respiratory complications, multiorgan failure, and in severe cases, death when improperly handled or administered [9,10].

There are inherent limitations to our reports; the study uses data and relies on records from medico-legal toxicology that may have insufficient clinical information. Non-fatal cases of plant intoxication were not included, reducing the possibility of comparison between fatal and non-fatal cases. The small number of cases limits the generalizability of the results.

These findings emphasize that the term “natural” is not necessarily associated with safety and highlight the need to increase awareness and education among clinicians, traditional practitioners, and the general public about the potential toxicity and dose-related risks of medicinal plants to prevent severe and sometimes fatal poisonings.

## References

[1] Latif R, Nawaz T (2025). Medicinal plants and human health: a comprehensive review of bioactive compounds, therapeutic effects, and applications. Phytochem Rev.

[2] Ouederni ARE, Omrani R, Ouederni NE, Ouederni YE, Katar A, Amdouni N (2024). A field Exploration and Inventory of the Most Toxic Plants of Southern Flora Tunisian. Indian J Pharm Educ Res.

[3] Hamouda C, Amamou M, Thabet H, Yacoub M, Hedhili A, Bescharnia F (2000). Plant poisonings from herbal medication admitted to a Tunisian toxicologic intensive care unit, 1983-1998. Vet Hum Toxicol.

[4] Wyk A, Prinsloo G (2020). Health, safety and quality concerns of plant-based traditional medicines and herbal remedies. S Afr J Bot.

[5] Achour S, Rhalem N, Khattabi A, Lotfi H, Mokhtari A, Soulaymani A (2012). *Peganum harmala* L. poisoning in Morocco: about 200 cases. Therapie.

[6] Li LN (2024). *Peganum harmala* A Review of Botany, Traditional Use, Phytochemistry, Pharmacology, Quality Marker, and Toxicity. Comb Chem High Throughput Screen.

[7] Tang S, Ren J, Kong L, Yan G, Liu C, Han Y (2023). Ephedrae Herba: A Review of Its Phytochemistry, Pharmacology, Clinical Application, and Alkaloid Toxicity. Molecules.

[8] Haller CA, Benowitz NL (2000). Adverse cardiovascular and central nervous system events associated with dietary supplements containing ephedra alkaloids. N Engl J Med.

[9] Anzano A, Falco Bd, Grauso L, Lanzotti V (2024). Squirting Cucumber, *Ecballium elaterium* (L.) A Ritch: An Update of Its Chemical and Pharmacological Profile. Molecules.

[10] Brouzas D, Oanta M, Loukianou E, Moschos M (2012). Keratoconjunctivitis and Periorbital Edema due to *Ecballium elaterium*. Case Rep Ophthalmol.

